# Legacy Effects Overshadow Tree Diversity Effects on Soil Fungal Communities in Oil Palm-Enrichment Plantations

**DOI:** 10.3390/microorganisms8101577

**Published:** 2020-10-13

**Authors:** Johannes Ballauff, Delphine Clara Zemp, Dominik Schneider, Bambang Irawan, Rolf Daniel, Andrea Polle

**Affiliations:** 1 Forest Botany and Tree Physiology, University of Goettingen, Büsgenweg 2, 37077 Göttingen, Germany; apolle@gwdg.de; 2 Biodiversity, Macroecology and Biogeography, University of Goettingen, Büsgenweg 1, 37077 Göttingen, Germany; delphine-clara.zemp@forst.uni-goettingen.de; 3 Genomic and Applied Microbiology and Göttingen Genomics Laboratory, University of Goettingen, 37077 Göttingen, Germany; dschnei1@gwdg.de (D.S.); rdaniel@gwdg.de (R.D.); 4 Faculty of Forestry, University of Jambi, Jln Raya Jambi-Ma.Bulian KM 15 Mendalo Darat Kode Pos, Jambi 36361, Indonesia; irawanbam@yahoo.com

**Keywords:** fungi, soil fungi, mycorrhiza, saprotrophic fungi, pathogenic fungi, agroforestry, oil palm, biodiversity, structure, tree diversity experiment

## Abstract

Financially profitable large-scale cultivation of oil palm monocultures in previously diverse tropical rain forest areas constitutes a major ecological crisis today. Not only is a large proportion of the aboveground diversity lost, but the belowground soil microbiome, which is important for the sustainability of soil function, is massively altered. Intermixing oil palms with native tree species promotes vegetation biodiversity and stand structural complexity in plantations, but the impact on soil fungi remains unknown. Here, we analyzed the diversity and community composition of soil fungi three years after tree diversity enrichment in an oil palm plantation in Sumatra (Indonesia). We tested the effects of tree diversity, stand structural complexity indices, and soil abiotic conditions on the diversity and community composition of soil fungi. We hypothesized that the enrichment experiment alters the taxonomic and functional community composition, promoting soil fungal diversity. Fungal community composition was affected by soil abiotic conditions (pH, N, and P), but not by tree diversity and stand structural complexity indices. These results suggest that intensive land use and abiotic filters are a legacy to fungal communities, overshadowing the structuring effects of the vegetation, at least in the initial years after enrichment plantings.

## 1. Introduction

There is a global increasing demand for vegetable oil. Oil palms have a superior yield potential compared to any alternative crops to meet market requirements [1,2]. Especially in Indonesia, the world’s biggest exporter of palm oil, this industry has led to a tremendous economic uprising of the country [3,4,5]. On the downside of these positive effects is the conversion of hyper-diverse tropical rainforest into tree mono-plantations, driving the loss of forest cover in South East Asia in the last decades [6,7,8,9,10]. The heavily managed oil palm monocultures only harbor a small proportion of the naturally occurring plant and animal biodiversity [11,12,13]. A wide range of ecosystem services is massively impaired [14,15,16]. It is, therefore, crucial to find alternative management strategies suitable to maintain profitability, while at the same time sustaining local biodiversity and ecosystem functions [17,18]. Agroforestry systems with intermixed tree and crop species are a promising approach to find a balance between economic productivity and ecosystem functions of tropical systems [19,20,21,22,23].

Soil-borne fungi are a key component of all terrestrial ecosystems facilitating nutrient flow and contributing to plant performance and health [24,25,26]. The diversity and composition of these communities are driven by abiotic soil variables, especially pH and soil nutrients [27,28,29,30,31]. However, vegetation and belowground fungal communities form numerous interactions and are strongly dependent on each other [32,33,34]. Thus, changes in both edaphic properties and plant community composition drive changes in fungal communities across tropical agroforestry systems [35,36]. Transformation of forests to oil-palm plantations strongly alters the soil fungal community [28,37,38], negatively affecting root health abundance of mycorrhizas while promoting plant pathogenic fungi [28,39]. Management strategies counteracting those shifts may promote fungal diversity and, thus, contribute to ecosystem restoration [40,41]. Enriching oil palm plantations with various tree species could allow to restore ecosystem functions and biodiversity, while at the same time providing socioeconomic benefits [19]. If and how tree diversity enrichment in previously intensively managed oil palm plantations affects soil fungal communities has not been studied.

Here, we investigated the soil fungal community composition in the enrichment experiment *EFForTS-BEE* located in the province Jambi (Sumatra, Indonesia). The experiment is designed to test whether mixed-species tree planting and natural regeneration are suitable strategies to restore biodiversity and ecosystem functions in existing oil palm plantations [42]. Native multipurpose tree species were planted within plots of varying sizes, tree species compositions, and diversity levels, established in a large-scale commercial oil palm plantation. Three years after establishment of the experiment, tree diversity significantly enhanced stand structural complexity [43]. The fastest growing tree species reached a height of approximately 6 m with a stem basal area of more than 20 cm^2^, while slow-growing species were well below the canopy with heights of 2.3 m and stem basal areas of only 2.7 cm^2^ [44].

We studied soil fungal communities using next-generation sequencing three years after establishment of the experiment. We measured soil pH, as well as carbon, nitrogen, and available phosphorus concentrations, to control for their structuring effect on the fungal community. We determined the effects of the experimental treatment and edaphic conditions on the soil fungal community composition to test the following hypotheses: (i) tree diversity enrichment alters soil fungal communities, promoting plant beneficial mycorrhizal fungi, thus mitigating the structuring effect of intensive land use; (ii) individual tree species distinctly shape the soil fungal community due to specific plant fungus interactions.

## 2. Material and Methods

### 2.1. Research Site and Experimental Design

This study was conducted in the framework of the biodiversity enrichment experiment *EFForTS-BEE* of the Collaborative Research Center 990 [42,44]. The research site is located in a commercial oil palm plantation (PT Humusindo Makmur Sejati, 01.95 south (S) and 103.25 east (E), 47 ± 11 m above sea level (a.s.l.)) on Sumatra (Indonesia). The climate is humid tropical (mean annual temperature: 26.7 ± 1.0 °C, annual precipitation: 2235 ± 285 mm) [11] on loamy Acrisol soil [45]. Dipterocarp-dominated low-land rainforests were the primary natural vegetation before transformation [8]. The oil palm plantation was established between 2001 and 2007. The oil palms were planted in a 9 m × 9 m triangular grid. Common management practices in oil palm plantations result in three distinct areas with widely varying conditions: interrow paths frequently used by workers and small vehicles such as motorbikes, frond piles of cut oil palm fronds, and management zones around each oil palm, where fertilizer is applied. Conventional management comprised the application of 230 kg N (urea), 196 kg P (triple superphosphate, rock phosphate), 142 kg K (KCl), 54 kg Mg (kiserite, dolomite), 0.79 kg B (borax) per ha and year, and the occasional addition of S ((NH_4_)_2_SO_4_), Si (zeolite), and Ca [42]. Furthermore, the plantation was regularly manually weeded [42]. In the plantation of 140 ha, 52 tree enrichment plots of varying size and tree diversity were established in December 2013. The details were described in Teuscher et al. [42], and a schematic overview of the experimental and sampling design is provided in Figure S1 (Supplementary Materials). The experimental design follows the random partition design described by Bell et al. [46], which allows for the independent testing of tree species identity and richness effects. In short, enrichment plots had sizes of 5 m × 5 m, 10 m × 10 m, 20 m × 20 m, and 40 m × 40 m. In the plots, the oil palms were thinned by about 40%. The number of felled oil palms depended on the plot size [47]. The following tree species were planted: *Parkia speciosa* (Fabaceae), *Archidendron pauciflorum* (Fabaceae), *Durio zibethinus* (Malvaceae), *Peronema canescens* (Lamiaceae), *Shorea leprosula* (Dipterocarpaceae), and *Dyera polyphylla* (Apocynaceae). Trees were planted in five different diversity levels (0, 1, 2, 3, and 6 species per plot). Each diversity level was repeated four times with different plot sizes (Table S1, Supplementary Materials). Combinations of two or three tree species were drawn at random with the restriction that no repetition was allowed and that each species was selected exactly once at each diversity level (Table S1, Supplementary Materials). Plots were distributed randomly on the plantation by maximizing the distance between them. The trees were planted in a 2 m grid between the oil palms. Newly planted trees were initially fertilized once (19 kg N, 8 kg P, 6 kg Mg; organic: 11 kg N, 7 kg P, 10 kg K, 4 kg Mg, 20 kg Ca; amounts are per ha). Mechanical weeding around the tree base was conducted initially to prevent overgrowth by understory vegetation. In May 2016, treatments were stopped to allow for natural succession. Four additional plots with management as usual (no removal of oil palm, usual fertilization, weeding, and harvesting) in the plantation were included as control plots. In total, the experiment comprised 56 research plots.

### 2.2. Sampling

Sampling was conducted in December 2016, two months after the beginning of the regional rainy season. In each research plot, three soil cores (10 cm depth, 4 cm diameter) were extracted in a 5 × 5 m area regardless of plot size to avoid effects of spatial distance, resulting in a total of 3 × 56 = 168 samples (Figure S1C, Supplementary Materials). In the control plots with the usual management, soil samples were only taken in the management zones. This was done to avoid additional variability caused by the widely differing conditions among management zones, interrow paths, and frond stacks. The latter two zones do not exist in the enriched plots and, therefore, were excluded for this study. The minimum distance to the plot border was 2 m. Samples were sieved (5 × 5 mm mesh), and roots and litter were removed. Aliquots of soil samples were immediately freeze-dried (VirTis Bench Top K, SP Industries, Warminster, PA, USA) and exported to the University of Göttingen (Germany). Sampling and export permission numbers by the Indonesian authorities are stated in Appendix A.

### 2.3. Soil Nutrient Elements and pH

Dry soil samples were ground for 1 min in a ball-mill (MM 2000, Retsch, Haan, Germany). Soil samples were weighed into tin cartouches and used to determine total carbon (C) and nitrogen (N) via the combustion method in a C/N analyzer (Vario MICRO analyzer, Elementar, Langensbold, Germany). Plant available phosphorus (P) was extracted following the method of Bray and Kurtz [48]. Soil (100 mg) was mixed with 15 mL of Bray-I Extraction Solution (0.03 N NH_4_F and 0.025 N HCl). The suspension was placed on a shaker for 60 min and subsequently filtered through phosphate free filters (MN 280 1/4 125 mm, Macherey–Nagel, Düren, Germany). Phosphate concentration of the filtrates was measured by inductively coupled plasma mass spectrometry (iCap 7000, Thermo Fisher Scientific, Waltham, MA, USA). Soil pH was measured in a 0.1 M KCl suspension according to ISO 10390 standard. The pH analysis was conducted in the Department of Soil Sciences of Temperate Ecosystems, Georg-August-University (Goettingen, Germany).

### 2.4. Vegetation Structure Complexity

To analyze the effect of modified vegetation structure in the enrichment plots, we included several measures of structural complexity derived from laser scanning in our analysis. All measurements were previously published by Zemp et al. [43] and detailed method descriptions can be found there. In brief, laser scans of the vegetation were carried out in the center of each plot and a field view of 360° horizontally and 300° vertically using a FARO Focus terrestrial laser scanner (Faro Technologies Inc., Lake Marry, FL, USA). Scanning took place in September and October 2016. On the basis of the laser scans, components of stand structural complexity above 1.3 m height were calculated following Ehbrecht et al. [49], using the mean fractal dimension of polygons resulting from cross-sections in the point cloud (MeanFRAC) and the effective number of layers (ENL) occupied by the vegetation [49,50]. ENL, thus, increases with stand height and evenly distributed vegetation across vertical layers [50] and is associated with the size of the oil palms in the *EFForTS-BEE* site [43], while MeanFRAC mainly depends on the vegetation density in three-dimensional space [49] and is rather associated with the diversity and performance of the planted tree species in the *EFForTS-BEE* site [43]. Finally, the stand structural complexity index (SSCI) is calculated by scaling MeanFRAC and ENL, thus integrating both measurements into an indicator of the structural complexity of the vegetation above 1.3 m height [49]. In addition, we computed the understory complexity index (UCI) according to the fractal dimension of horizontal “slices” of the point cloud between 0.8 and 1.8 m height projected on a horizontal plane, thereby reflecting the structure of the understory vegetation [51].

### 2.5. Fungal Community

DNA was extracted from 200 mg of freeze-dried soil of each of the 168 samples using the DNeasy PowerSoil Kit (Qiagen, Venlo, The Netherlands), following the manufacturer’s instructions. Subsequently, an additional clean-up step using the DNeasy PowerClean Cleanup Kit (Qiagen, Venlo, The Netherlands) was performed. Further purification, amplification, and library production were described elsewhere [52]. Barcoding of the fungal community was based on the internal transcript spacer region 2 (ITS2). The marker was amplified by polymerase chain reaction (PCR) using ITS3_KYO1 [53] and ITS4 [54]. Amplicon sequencing was conducted at the Göttingen Genomics Laboratory with the MiSeq Reagent Kit v3 and the MiSeq platform as recommended by the manufacturer (Illumina Inc., San Diego, CA, USA).

The raw paired ends were merged with PEAR v.0.9.10 [55] and quality filtering was performed with Trimmomatic v.0.36 [56]. Cutadapt v.1.16 [57] was used to clip the primer sequences. Reads with a length < 140 bp were excluded, and VSEARCH v.2.7.2 [58] was used for dereplication, denoising (removal of reads with fewer than eight occurrences), and chimera detection (de novo followed by reference-based). The resulting amplicon sequence variants (ASVs) were used to generate operational taxonomic units (OTUs) by clustering at a 97% similarity threshold. Taxonomic assignment of OTUs was carried out using the representative sequence of each OTU against the UNITE database v7.2 (UNITE_public_01.12.2017.fasta) [59] with the BLAST search algorithm (blastn, v2.7.1) [60]. All unidentified OTUs were searched (blastn, v2.7.1) [60] against the nt database of the National Center for Biotechnology Information (NCBI, January 2018) [61] to remove non-fungal OTUs; only fungal sequence reads were kept. Fungal OTUs were assigned to trophic guilds by taxonomy, according to the FunGuild database [62] (Table S2, Supplementary Materials). All reads were mapped to the resulting OTU library to generate a count table.

The OTU count table was rarefied to the count number of 19,000 (minimum in one sample) using the *rrarefy()* function of the package vegan v2.5.6 [63]. Subsequently, counts of three samples per plot were added, resulting in 56 samples that represent the plot community.

### 2.6. Sequence Data Deposition

The ITS2 region sequences were deposited in the National Center for Biotechnology Information (NCBI) Sequence Read Archive (SRA) [64] under bioproject accession number PRJNA659225.

### 2.7. Statistical Analysis

Statistical analyses were performed using R v.3.6.1 [65]. Means of C, N, and P (*n* = 3 per plot), C/N ratio, and pH were used for subsequent analysis (Table S1, Supplementary Materials). Carbon and nitrogen content were highly correlated (*r* > 0.9). Total carbon was excluded from further analysis to avoid multicollinearity.

The fungal diversity was accessed using the Hill numbers framework [66]. The function *hill_taxa()* of the package hillR [67] was used to calculate taxonomic diversity at the OTU level using the dimensions 0 (^0^D), corresponding to OTU richness, and 1 (^1^D), corresponding to the Shannon entropy. A phylogenetic tree was constructed from the aligned OTU sequences, and the phylogenetic diversity was calculated using the *hill_phylo()* function [67] with the dimensions 0 and 1.

Linear regression models were fitted to analyze the effect of tree species enrichment on fungal diversity indices. The *anova()* function [65] was used to test overall significance of the fitted models. Linear regression models were also used to evaluate the effect of soil variables (pH, N, C/N, P), as well as stand structure (SSCI, ENL, MeanFRAC, UCI), on fungal diversity.

The relative importance of tree richness, tree species identity, and plot size was quantified using a linear model for random partition design [46] that was adapted to the design of *EFForTS-BEE* [42,44]. This design includes terms for tree species richness treated as a continuous variable (i.e., 0, 1, 2, 3, or 6), a numeric matrix indicating the presence or absence of each tree species, the number of planted tree species as a discrete variable (“nonlinear species richness”), and plot size. The model allows for independent testing of species identity effects and species interaction effects (represented by the nonlinear richness term). Furthermore, the contribution of individual species can be analyzed by directly comparing their associated coefficients. To control for the structuring effects of soil abiotic variables, pH, N, C/N, and P were also included in the model. The importance of all variables was estimated using the mean square of the respective coefficient in an analysis of variance (ANOVA) in a sequential manner—using the residuals as explanatory variable for the next model—in the order mentioned above. The pH gradient across the plots varied from 3.75 to 4.56 units; however, in four plots (plot No. 2, 24, 31, and 46; see Table S1, Supplementary Materials), extreme outliers (range: 5.14 to 6.32, >1.5× interquartile range) were present, potentially strongly determining the model parameters. Likewise, in plot No. 46, a very high nitrogen concentration (3.62 mg·g^−1^, >1.5× interquartile range) compared to the remaining values ranging from 1.03 to 2.89 mg·g^−1^ was detected. To evaluate the effects of those outliers on the model estimates, all models were recalculated after omitting those data points and imputing them with the respective median of the whole dataset. However, overall trends of the results were not affected by the extreme values (Table 2, Table S3, Supplementary Materials).

Effects of tree richness on the community composition of fungal taxa (genus, family, and order level) and trophic groups (saprotroph, pathotroph, symbiotroph) were tested using analysis of similarity (ANOSIM) as implemented in the *anosim()* function [63]. To estimate *p*-values, 999 permutations were calculated. Likewise, the effect of tree diversity on the fungal OTU community composition was tested using the *anosim()* function [63] with 999 permutations. Fungal community composition was further explored using principle coordinate analysis (PCoA) as implemented in the *cmdscale()* function [63]. Structuring effects of soil pH, N, C/N, and P, as well as vegetation SSCI, ENL, MeanFRAC, and UCI, were analyzed using the *envfit()* function [63].

We further calculated the a distance-based redundancy analysis (db-RDA) as implemented in vegan’s *cca()* function [63] for the random partition design, including terms for soil variables, as well as tree richness, species identity, nonlinear tree richness, and plot size. The *anova.cca()* function [63] was used to subsequently calculate an ANOVA-like permutation test (999 permutations) for each term. To estimate the contribution of all tree species (*P. speciosa*, *A. pauciflorum*, *D. zibethinus*, *P. canescens*, *S. leprosula*, *D. polyphylla*) to the fungal community structure, we first calculated a model only including soil parameters and tree richness, and we subsequently calculated a model including the tree species matrix as the independent variable and the residuals of the previous model as the dependent variable. Associated canonical coefficients for all tree species were obtained from this model. The significance for each marginal term was tested using *anova.cca()* [63] with 999 permutations.

## 3. Results

We obtained a total number of 8283 OTUs in the rarefied dataset (total number sequence counts: ~3.1 million), with a mean richness of 1501 (±220) per plot. Fungal taxonomic and phylogenetic diversity in tree plots did not differ from the control plantation sites regardless of tree diversity level (Table 1). We found a higher soil C/N ratio in control sites compared to tree enrichment plots, while no effects on other soil abiotic variables (pH, N, P) were observed (Table 1).

**Figure 1:**
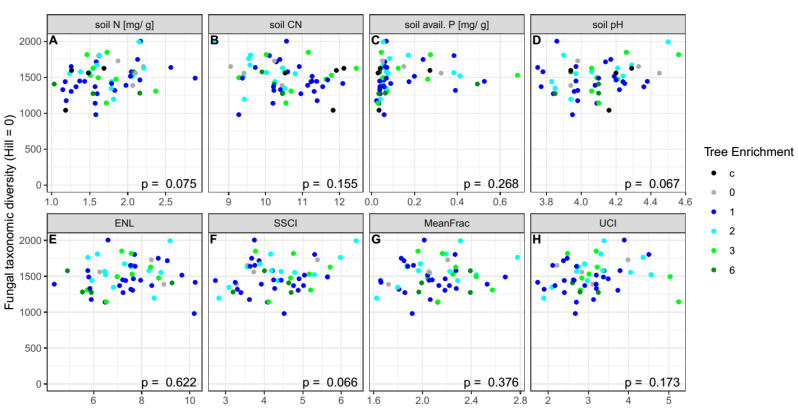
Relationship between fungal taxonomic diversity (expressed as Hill number with dimension 0) and soil abiotic variables ((**A**): nitrogen, (**B**): carbon-to-nitrogen ratio, (**C**): available phosphorus, (**D**): pH), as well as different components of vegetation structural complexity ((**E**): effective number of layers (ENL), (**F**): stand structural complexity index (SSCI), (**G**): mean fractal dimension of polygons resulting from cross-sections in the point cloud (MeanFRAC), (**H**): understory complexity index (UCI)). N: nitrogen, C/N: carbon-to-nitrogen ratio, P: phosphorus. Linear models were fitted to the data. No significant relationships (F-test in ANOVA, *p*-value < 0.05) were found. Colors denote tree richness; c: oil palm control plots with management as usual.

To test the linear effects of soil variables, as well as vegetation stand complexity (ENL, MeanFRAC, SSCI, and UCI), we constructed linear regression models with Hill numbers of taxonomic and phylogenetic functional diversity (Figure 1, Figures S2–S4, Supplementary Materials). No significant effects of soil pH (F_1,50_ = 3.494, *p* = 0.067), soil nitrogen (F_1,53_ = 3.307, *p* = 0.075), soil C/N (F_1,54_ = 2.081, *p* = 0.155), and available phosphorus (F_1,54_ = 1.253, *p* = 0.268) on fungal OTU richness (^0^D) were found (Figure 1A–D). Likewise, no effects of stand structure measures were observed (SSCI: F_1,50_ = 3.534, *p* = 0.066; ENL: F_1,50_ = 0.246, *p* = 0.622; MeanFRAC: F_1,50_ = 0.799, *p* = 0.376; UCI: F_1,50_ = 1.913, *p* = 0.172; Figure 1E–G). Similar results were obtained for the phylogenetic diversity and higher-order Hill numbers (Figures S2–S4, Supplementary Materials). Notably, increased soil pH positively affected fungal phylogenetic diversity (^0^D: F_1,50_ = 4.131, *p* = 0.047), and UCI had a positive effect on higher-order taxonomic Hill diversity (^1^D: F_1,50_ = 4.478, *p* = 0.039).

To investigate whether tree identity or tree diversity affected soil fungal diversity, we applied a linear model for random partitioning design, including soil properties, linear tree richness, a tree species identity matrix, and enrichment plot size. The model did not include the four control plots, because they were not part of the experimental treatment. In this model, soil pH was significant, affecting fungal taxonomic and phylogenetic diversity at ^0^D, while no significant effects were observed at higher-order Hill numbers (Table 2). Other soil variables, as well as tree richness, species identity, nonlinear tree richness, and plot size, had no significant effect (Table 2).

**Figure 2:**
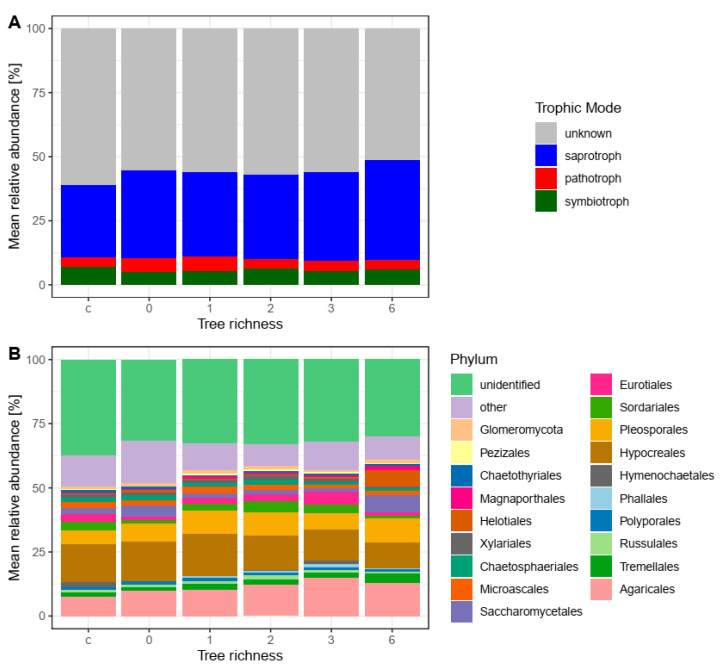
Mean relative abundance of fungal orders (**A**) and trophic groups (**B**) in enrichment plots with 0–6 additional planted tree species and in control plots (c) of oil palm with management as usual.

We further analyzed the structuring effects of tree species enrichment and stand structural complexity on the fungal community composition. The most abundant fungal phyla were Ascomycota (45.4%) followed by Basidiomycota (20.9%). Approximately 72.0% of all reads were taxonomically identified at the order level (Table S2, Supplementary Materials; Figure 2A). The most abundant orders were Hypocreales (Ascomycota), Pleosporales (Ascomycota), and Agaricales (Basidiomycota).

No compositional change among fungal orders was observed between different tree richness levels and conventionally managed sites (ANOSIM: *R* = 0.091; *p* = 0.127; Figure 2A). Likewise, we found no structuring effect of tree richness on fungal community composition at the taxonomic levels of family (ANOSIM: *R* = 0.054; *p* = 0.237) or genus (ANOSIM: *R* = 0.037; *p* = 0.314). Approximately 44% of the fungal reads could be assigned to a trophic mode (Table S2, Supplementary Materials; Figure 2B). We did not observe changes in the composition of trophic modes in the fungal communities among different levels of tree richness (ANOSIM: *R* = −0.06; *p* = 0.862, Figure 2B). Saprotrophic fungi accounted for approximately 50% of functionally annotated fungi (Table S1, Supplementary Materials) about 1% were litter and 1% dung saprotrophs; however, for most of the saprotrophs, substrates were not known (Table S1, Supplementary Materials).

**Figure 3:**
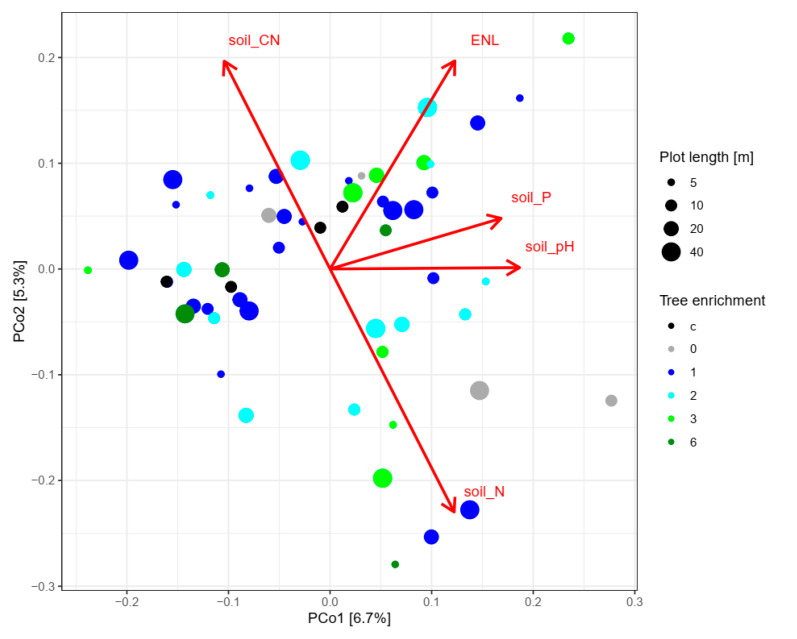
Principal coordinate analysis (PCoA) of fungal OTU community Bray–Curtis dissimilarity. Colors denote the number of planted tree species and point sizes indicate the size of enrichment plots. Red arrows show the significant linear effect of environmental variables (*p* < 0.05) associated with the ordination axes. N: nitrogen, C/N: carbon-to-nitrogen ratio, P: phosphorus, ENL: effective number of layers, c: oil palm control plots with management as usual.

No structuring effect by tree diversity on the fungal OTU community was observed (ANOSIM: *R* = −0.024; *p* = 0.655; Figure 3). Soil pH, nitrogen concentration, C/N ratio, and phosphorus concentration were significantly associated with the fungal community composition (Figure 3). Notably, we also observed a significant association of vegetation ENL with the fungal community composition (Figure 3).

To test the structuring effect of tree enrichment and tree species identity on the soil fungal community composition, we calculated a distance-based redundancy analysis for the random partition design. Effects of soil pH, as well as nitrogen and phosphorus concentration, on the structure of the fungal community were observed; however, there were no significant effects of tree richness, tree species identity, or plot size (Table 3). The contribution of individual tree species to the fungal community composition was analyzed by calculating the canonical coefficients associated with each tree species in the partial model. However, none of the coefficients was significant (Figure S5, Supplementary Materials).

## 4. Discussion

Soil fungi play a crucial role in the recovery of ecosystems [40,41]; however, only few studies addressed fungal communities in tropical forest restoration systems [68,69]. To our knowledge, this study is the first to explore possibilities to restore fungal communities in intensively managed oil palm plantations. Tree enrichment did not alter the soil fungal diversity or community composition compared to the surrounding plantation three years after tree planting and undergrowth succession. Mainly soil pH, as well as nitrogen and phosphorus concentrations, affected the fungal community in the studied plots, while few effects due to the elevated vegetation stand structural complexity in the enrichment plots were observed.

Soil fungal diversity was neither affected by the experimental treatment in the enrichment plots versus the control plots nor by increasing tree species richness. Transformation of tropical forest to oil palm and rubber monoculture had little effect on fungal OTU richness compared to the massive loss of plant diversity [70]. Therefore, we did not expect drastic changes in fungal richness driven by tree diversity enrichment, but rather a shift in their community composition. Previous studies showed that fungal community structures were altered in tropical land-transformation systems compared to rainforests [28,37,38]. Since neither the taxonomic nor the functional composition of the fungal community was affected by tree enrichment (compared to conventional management) or increased tree diversity, our first hypothesis that tree diversity enrichment alters soil fungal communities has to be rejected. Furthermore, we did not find any structuring effect of tree species identity. These results were surprising, because links between the plant composition and fungal community have frequently been observed and causal plant fungus relationships are generally assumed due to their numerous interactions [30,71,72,73]. Long-lasting DNA fragments from a dead, inactive fungal community may have been present in the soil. However, it is unlikely that they masked fungal turnover because numerous previous studies detected alterations in fungal community compositions, for example, in response to trenching, seasonal variation, and temporal turnover within time periods of 12–24 months [74,75,76].

The effective number of layers (ENL) in the vegetation structure was the only vegetation variable significantly associated with the composition of the soil fungal community. This index typically describes the vertical distribution of vegetation layers and increases with higher stand height, as well as a more equal distribution of vegetation layers [50]. In this enrichment experiment, however, ENL was strongly determined by the growth height and crown size of the oil palms [43]. This suggests that the fungal community may be structured by the oil palm growth (i.e., the dominating plant in the surrounding monoculture and in the enrichment plots prior to establishment of the experiment).

Our results suggest that there is a strong legacy effect on the fungal community, still overshadowing the structuring effects by the diverse plant community. In agreement to our results, long-lasting land-use effects on the soil fungal community composition were demonstrated even after 80 years of natural succession from farming and logging in tropical forests of Puerto Rico [68]. Furthermore, Chai et al. [77] demonstrated that significant compositional differences of the fungal community between managed and recovering sites were only apparent after more than a decade of natural succession from farmland to secondary forest. No differences were found in early establishment phases up to 15 years after stopping management [77]. Agroforestry systems promote compositional shifts in the fungal community and abundance of mycorrhizal fungi compared to monocultures for a variety of tropical crops, including coffee [78], cacao [35], and rubber [28]. Therefore, despite our first results, we expect that long-term establishment of mixed-species plots could lead to changes in the soil fungal communities and potentially promote the abundance of mycorrhizal fungi [28]. In particular, the presence of ectomycorrhiza-forming Dipterocarpaceae such as *Shorea leprosula* could contribute to the new establishment of ectomycorrhizal species over time. As most tree diversity experiments worldwide [79], *EFForTS-BEE* is still a relatively young experiment, and long-term research (e.g., several decades) is necessary to better understand how successional trajectory after intensive land use affects the fungal community.

Soil abiotic drivers heavily structure the fungal communities in soil and can overshadow potential plant regulatory effects [80,81]. In our study, relatively homogeneous conditions were found for soil variables typically driving soil fungal community composition [31,52,82,83]. Therefore, it was not surprising that only a low proportion of community variation was explained here by our models. Microbial communities are shaped by deterministic (e.g., environmental filtering) and niche-neutral processes such as dispersal limitation and stochastic community variation [28,84,85,86]. Ecologic drift (i.e., random population fluctuations) can dominate community assembly under homogeneous environmental conditions [87]. Similar mechanisms may have strongly contributed to community variation in our study. Soil pH and nutrient concentration (nitrogen and available phosphorus) partially determined the soil fungal community composition in our study, despite the flat environmental gradient. Soil pH influences the availability of nutrient elements, as well as toxic cations [29,39,88,89]. Therefore, soil pH is an important driver of belowground microbiomes [90,91]. Structuring effects of pH on the fungal community were demonstrated across steep gradients from acidic to basic soils [31,92], as well as smaller regional variations by one or two units [29,93,94]. Low nutrient concentrations, particularly phosphorus, are common in highly weathered tropical soils [45,95,96]. Therefore, P and N availability are likely limiting factors for soil microorganisms; consequently, tight associations between soil nutrients and community composition occur frequently [27,30,97,98]. However, the shifts in fungal communities are unrelated to root N uptake, which is similar in rainforests to that in oil palm plantations [99].

## 5. Conclusions

In this study, we showed that short-term (three years) manipulation of the tree diversity and associated changes in vegetation structure were not sufficient to strongly impact the soil fungal community after stopping intensive management. However, this does not imply that effects of enrichment tree planting will have no long-term positive effects on the soil fungal microbiome. The root-associated microbiome often differs significantly from the surrounding soil communities, as a consequence of host plant properties [100,101,102]. Agroforestry systems can promote diversity, alter composition, and improve the resilience of root-associated fungal communities [103,104,105], and those effects may generate feedback on the surrounding soil after long-term establishment of tree enrichment. Studying further succession, as well as analysis of the root-associated communities of planted tree species, could help to predict future effects of tree diversity enrichment on the soil microbiome in monoculture plantations.

## Figures and Tables

**Table 1:** Fungal taxonomic (taxon.) and phylogenetic (phylo.) diversity expressed as Hill numbers with weights on operational taxonomic unit (OTU) abundance equal to zero (^0^D) and one (^1^D), as well as soil abiotic variables. Means (± SD) for conventionally managed oil palm plantations (control) and experimental plots with 0, 1, 2, 3, or 6 different tree species are shown. The *p*-values show the results of an ANOVA (nitrogen and phosphorus concentration was transformed by natural logarithm). *n* = 56, degrees of freedom = 50, *** *p* < 0.001.

	Control	0	1	2	3	6	*p*-Value
Fungal diversity							
taxon. (^0^D)	1459 (±139)	1584 (±74)	1460 (±46)	1591 (±61)	1532 (±84)	1386 (±71)	0.457
taxon. (^1^D)	162.7 (±26.3)	172.6 (±25.5)	178.9 (±9.5)	174.3 (±17.1)	180.7 (±9.8)	134.0 (±35.2)	0.681
phylo. (^0^D)	825 (±61)	844 (±31)	827 (±19)	874 (±26)	859 (±41)	778 (±28)	0.552
phylo. (^1^D)	39.6 (±2.4)	38.4 (±3.5)	43.2 (±1.5)	42.0 (±2.9)	45.1 (±2.0)	37.5 (±5.3)	0.552
Soil variables							
Soil pH	4.15 (±0.07)	4.18 (±0.13)	4.11 (±0.07)	4.42 (±0.24)	4.31 (±0.13)	4.00 (±0.06)	0.454
Soil nitrogen (mg·g^−1^)	1.40 (±0.11)	2.06 (±0.07)	1.72 (±0.09)	1.90 (±0.18)	1.72 (±0.10)	1.70 (±0.26)	0.277
Soil C/N	11.60 (±0.36)	9.79 (±0.33)	10.73 (±0.15)	9.90 (±0.18)	10.95 (±0.36)	10.26 (±0.08)	<0.001 ***
Soil phosphorus (mg·g^−1^)	0.09 (±0.06)	0.14 (±0.07)	0.12 (±0.03)	0.13 (±0.04)	0.19 (±0.08)	0.16 (±0.11)	0.854

**Table 2:** Effect of soil variables and tree enrichment on fungal diversity in the oil palm plantation. Hill numbers were calculated with weight on OTU abundance equal to zero (^0^D) and one (^1^D). Significant effects were tested using ANOVA. Df: degrees of freedom, Sq: square, C/N: carbon-to-nitrogen ratio, * *p* < 0.05. Nonlinear tree richness is an approximation for interactions among tree species, independent of their identity.

	Df	Sum Sq	Mean Sq	F-Value	*p*-Value
Taxonomic diversity (^0^D)					
Soil pH	1	226,996	226,996	5.1855	0.0290 *
Soil nitrogen (mg·g^−1^)	1	98,642	98,642	2.2534	0.1423
Soil C/N	1	18,090	18,090	0.4132	0.5245
Soil phosphorus (mg·g^−1^)	1	11,620	11,620	0.2655	0.6096
Linear tree richness	1	12,349	12,349	0.2821	0.5987
Tree species identity	5	361,799	72,360	1.653	0.1719
Nonlinear tree richness	3	106,425	35,475	0.8104	0.4967
Plot size	3	56,147	18,716	0.4275	0.7345
Residuals	35	1,532,123	43,775		
Taxonomic diversity (^1^D)					
Soil pH	1	14	13.6	0.0046	0.9462
Soil nitrogen (mg·g^−1^)	1	1302	1301.6	0.4414	0.5108
Soil C/N	1	1137	1136.7	0.3855	0.5387
Soil phosphorus (mg·g^−1^)	1	5841	5840.7	1.9808	0.1681
Linear tree richness	1	3681	3680.8	1.2483	0.2715
Tree species identity	5	1491	298.2	0.1011	0.9913
Nonlinear tree richness	3	2109	702.9	0.2384	0.869
Plot size	3	5329	1776.3	0.6024	0.6178
Residuals	35	103,205	2948.7		
Phylogenetic diversity (^0^D)					
Soil pH	1	56,524	56,524	6.6913	0.01401 *
Soil nitrogen (mg·g^−1^)	1	1987	1987	0.2353	0.63068
Soil C/N	1	553	553	0.0654	0.79963
Soil phosphorus (mg·g^−1^)	1	4	4	0.0004	0.98342
Linear tree richness	1	2657	2657	0.3145	0.57851
Tree species identity	5	60,943	12,189	1.4429	0.23345
Nonlinear tree richness	3	18,996	6332	0.7496	0.52997
Plot size	3	8775	2925	0.3463	0.79203
Residuals	35	295,657	8447		
Phylogenetic diversity (^1^D)					
Soil pH	1	0.07	0.074	0.001	0.9752
Soil nitrogen (mg·g^−1^)	1	142.1	142.098	1.8859	0.1784
Soil C/N	1	69.96	69.96	0.9285	0.3419
Soil phosphorus (mg·g^−1^)	1	124.3	124.304	1.6498	0.2074
Linear tree richness	1	15.12	15.116	0.2006	0.657
Tree species identity	5	114.49	22.897	0.3039	0.9072
Nonlinear tree richness	3	118.95	39.65	0.5262	0.6672
Plot size	3	81.17	27.058	0.3591	0.7829
Residuals	35	2637.12	75.346		

**Table 3:** ANOVA-like permutation test for distance-based redundancy analysis (dbRDA) of the fungal community composition (999 permutations). Df: degrees of freedom, Sq: square, C/N: carbon-to-nitrogen ratio, *** *p* < 0.001, ** *p* < 0.01, * *p* < 0.05. Nonlinear tree richness is an approximation for interactions among tree species, independent of their identity.

	Df	Sum Sq	F-Value	*p*-Value
Soil pH	1	0.2474	1.2965	0.017 *
Soil nitrogen (mg g^−1^)	1	0.3727	1.9533	0.001 ***
Soil C/N	1	0.2329	1.2204	0.063
Soil phosphorus (mg g^−1^)	1	0.2873	1.5054	0.003 **
Linear tree richness	1	0.1957	1.0255	0.381
Tree species identity	5	0.9462	0.9917	0.561
Nonlinear tree richness	3	0.5548	0.9692	0.657
Plot size	3	0.6293	1.0993	0.096
Residuals	35	6.6788		

## Supplementary Materials

The following are available online at https://www.mdpi.com/2076-2607/8/10/1577/s1, Figure S1: Adapted from Teuscher et al. (2016) in Frontiers in Plant Science (vol. 17). Figure S2: Relationship between fungal taxonomic diversity (Hill 1) and soil abiotic variables (A: nitrogen, B: carbon to nitrogen ratio, C: available phosphorus, D: pH) as well as vegetation structure indices (E: ENL, F: SSCI, G: MeanFRAC, H: UCI), Figure S3: Relationship between fungal phylogenetic diversity (Hill 0) and soil abiotic variables (A: nitrogen, B: carbon to nitrogen ratio, C: available phosphorus, D: pH) as well as vegetation structure indices (E: ENL, F: SSCI, G: MeanFRAC, H: UCI), Figure S4: Relationship between fungal phylogenetic diversity (Hill 1) and soil abiotic variables (A: nitrogen, B: carbon to nitrogen ratio, C: available phosphorus, D: pH) as well as vegetation structure indices (E: ENL, F: SSCI, G: MeanFRAC, H: UCI), Figure S5: Canonical coefficients of distance-based redundancy analysis (db-RDA) associated with the effect of each planted tree species on the fungal community composition in the model for the random partitioning. A permutation test (999 permutations) was used to calculate statistic significances. No significant effects were found, Table S1: Tree enrichment island size (edge length), tree species richness levels, planted tree species, soil chemical measurements and fungal OTU diversity measures, Table S2: Rarefied read counts of fungal operational taxonomic units (OTU; clustered at 97 % similarity threshold) for all tree islands and reference plots. (coluns 1–57) as well as their taxonomic, Table S3: Effect of soil variables and tree enrichment on fungal diversity in the oil palm plantation. Hill numbers were calculated with weight on OTU abundance equals zero (^0^D) and equals one (^1^D). Significant effects were tested using ANOVA. Outlier values of soil pH and nitrogen were replaced by their respective median values. Df: degree of freedom, Sq: square, CN: carbon to nitrogen ratio, *p*-value < 0.001 ‘***’, <0.01 ‘**’, <0.05 ‘*’. Non-linear tree richness is an approximation for interactions among tree species, independent of their identity.

## Appendix A. Sampling and Export Permission

Research permit (Surat Izin Penelitian, reference number: 328/SIP/FRP/E5/Dit.KI/IX/2016) was issued by the Ministry of Research Technology and Higher Education (Kementrian Riset, Teknologi dan Pendidikan Tinggi, Jakarta, Indonesia). The permit recommendations for sample collection, domestic sample transport, sample export, and access to the genetic material were issued by the Research Center for Biology of the Indonesian Institute of Science (Lembaga Ilmu Pengetahuan Indonesia, Jakarta, Indonesia), numbers 2781/IPH.1/KS.02.04/IX/2016 and B-1345/IPH.1/KS.02.04/III/2019.

The permit for sample collection, domestic sample transport, sample export, and access to the genetic material was issued by the Ministry of Environment and Forestry, Directorate General of Conservation of Natural Resources and Ecosystems of the Republic of Indonesia (Izin akses sumber daya genetik untuk kepentingan penelitian oleh Kementerian Lingkungan Hidup dan Kehutanan, Direktorat Jenderal Konservasi Sumber Daya Alam dan Ekosistem), numbers S.1068/KKH/SDG/KSA.2/11/2016 and SK.335/KSDAE/SET/KSA.2/7/2019.

The Chamber of Agriculture of Lower Saxony (Plant Protection Office, Hannover, Germany) issued the import permits (Letter of Authority, numbers: DE-NI-17-03 2008 -61-EC).
