# Supplementary material for: Legacy Effects Overshadow Tree Diversity Effects on Soil Fungal Communities in Oil Palm-Enrichment Plantations
Source: Microorganisms. 2020 Oct 13;8(10):1577. doi: 10.3390/microorganisms8101577 (PMC7656304; doi:10.3390/microorganisms8101577)
Supplement: Supplementary file 1 [file microorganisms-08-01577-s001.zip › suppl_proofread/microorganisms-933297-suyppl.docx]

Supplementary File

Legacy Effects Overshadow Tree Diversity Effects on Soil Fungal Communities in Oil Palm-Enrichment Plantations

Johannes Ballauff ^1,^*, Delphine Clara Zemp ^2^, Dominik Schneider ^3^, Bambang Irawan ^4^,
Rolf Daniel ^3^ and Andrea Polle ^1^

^1^ Forest Botany and Tree Physiology, University of Goettingen, Büsgenweg 2, 37077 Göttingen, Germany; apolle@gwdg.de

^2^ Biodiversity, Macroecology and Biogeography, University of Goettingen, Büsgenweg 1, 37077 Göttingen, Germany; delphine‑clara.zemp@forst.uni-goettingen.de

^3^ Genomic and Applied Microbiology and Göttingen Genomics Laboratory, University of Goettingen, 37077 Göttingen, Germany; dschnei1@gwdg.de (D.S.); rdaniel@gwdg.de (R.D.)

^4^ Faculty of Forestry, University of Jambi, Jln Raya Jambi-Ma.Bulian KM 15 Mendalo Darat Kode Pos, 36361, Indonesia; irawanbam@yahoo.com

***** Correspondence: jballau@gwdg.de


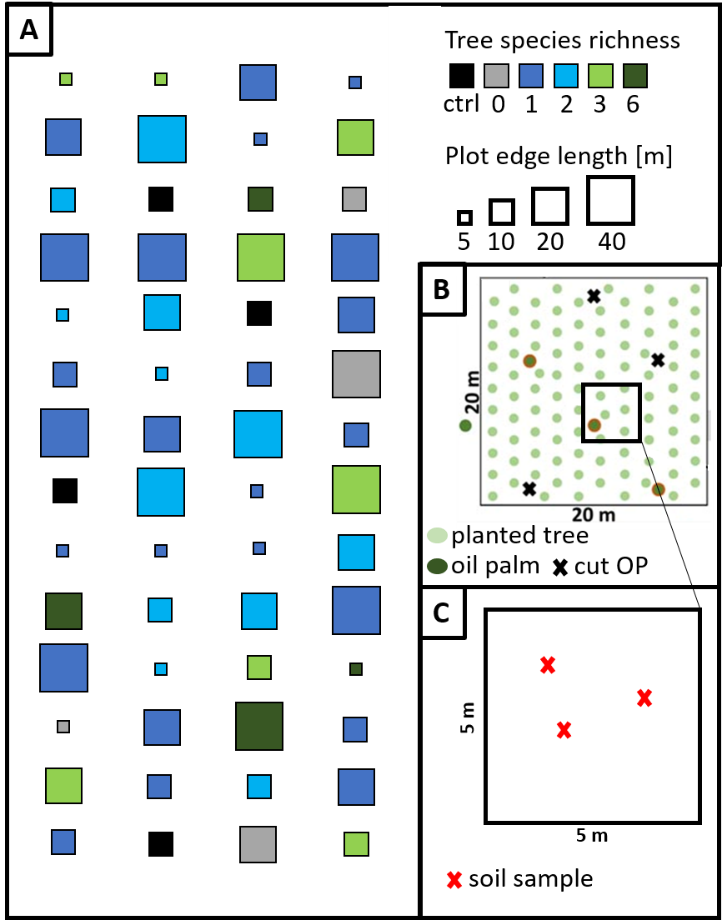


**Figure S1.** Adapted from Teuscher et al. (2016) in Frontiers in Plant Science (vol. 17). **A:** Design of the biodiversity enrichment experiment (EFForTS-BEE). Tree enrichment plots were established within a continuous oil palm plantation. They are varying in tree species richness (richness level 0, 1, 2, 3, 6 and usual management control plots), size (edge length 5, 10, 20, 40 m), tree species identities and compositions. *Parkia speciosa* (Fabaceae), *Archidendron pauciflorum* (Fabaceae), *Durio zibethinus* (Malvaceae), *Peronema canescens* (Lamiaceae), *Shorea leprosula* (Dipterocarpaceae) and *Dyera polyphylla* (Apocynaceae) were planted. At each level of tree richness each tree species is represented exactly once. **B:** Exemplary representation of one research plot with planted trees in a 2 × 2 m grid and cut oil palms (OP) for improved light conditions. Black square represents 5 × 5 m subplot were soil samples were collected. **C:** Three soil cores (depth 10 cm, diameter 4 cm) were collected in each 5 × 5 m subplot.


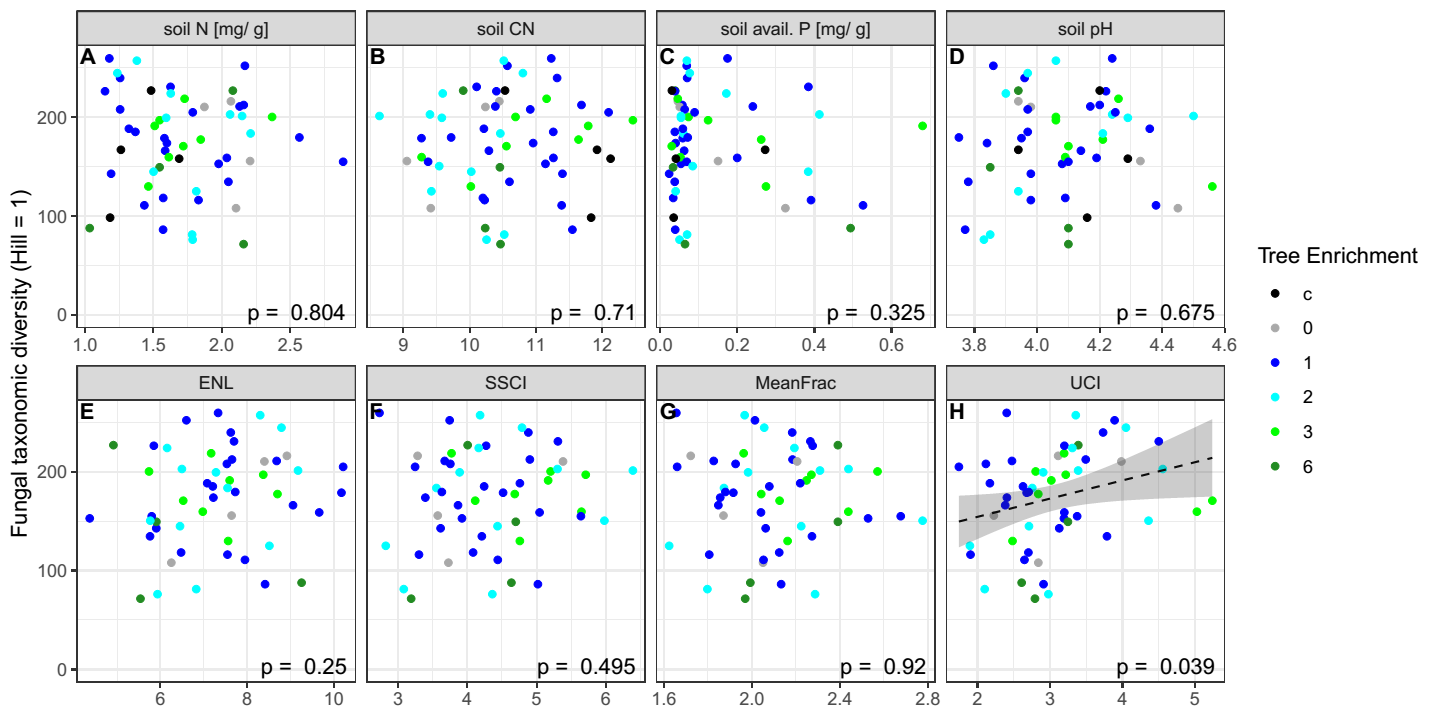


**Figure S2.** Relationship between fungal taxonomic diversity (expressed as Hill number with dimension 1) and soil abiotic variables ((**A**): nitrogen, (**B**): carbon-to-nitrogen ratio, (**C**): available phosphorus, (**D**): pH), as well as different components of vegetation structural complexity ((**E**): effective number of layers (ENL), (**F**): stand structural complexity index (SSCI), (**G**): mean fractal dimension of polygons resulting from cross-sections in the point cloud (MeanFRAC), (**H**): understory complexity index (UCI)). N: nitrogen, C/N: carbon-to-nitrogen ratio, P: phosphorus. Linear models were fitted to the data. Significant cases (F-test in ANOVA, p-value < 0.05) are shown. Colors denote tree richness; c: oil palm control plots with management as usual.


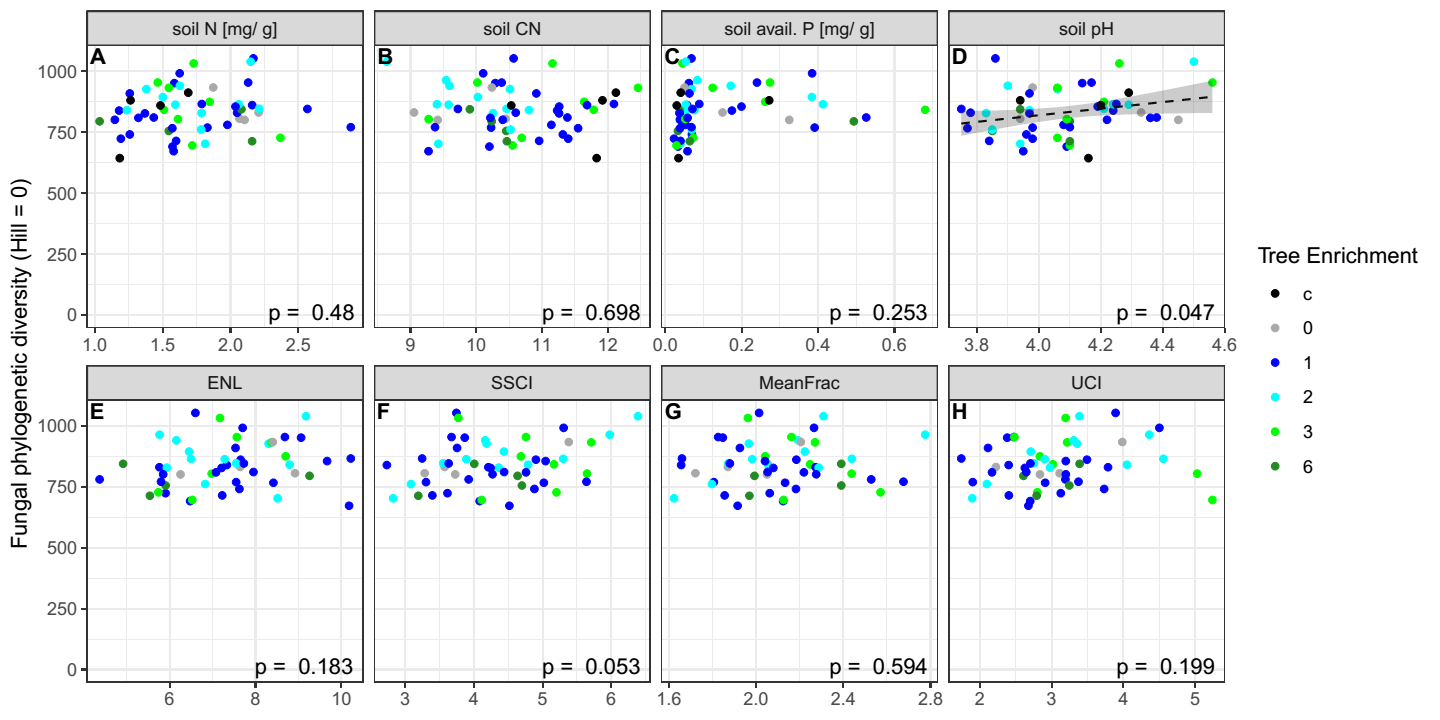


**Figure S3.** Relationship between fungal phylogenetic diversity (expressed as Hill number with dimension 0) and soil abiotic variables ((**A**): nitrogen, (**B**): carbon-to-nitrogen ratio, (**C**): available phosphorus, (**D**): pH), as well as different components of vegetation structural complexity ((**E**): effective number of layers (ENL), (**F**): stand structural complexity index (SSCI), (**G**): mean fractal dimension of polygons resulting from cross-sections in the point cloud (MeanFRAC), (**H**): understory complexity index (UCI)). N: nitrogen, C/N: carbon-to-nitrogen ratio, P: phosphorus. Linear models were fitted to the data. Significant cases (F-test in ANOVA, p-value < 0.05) are shown. Colors denote tree richness; c: oil palm control plots with management as usual.


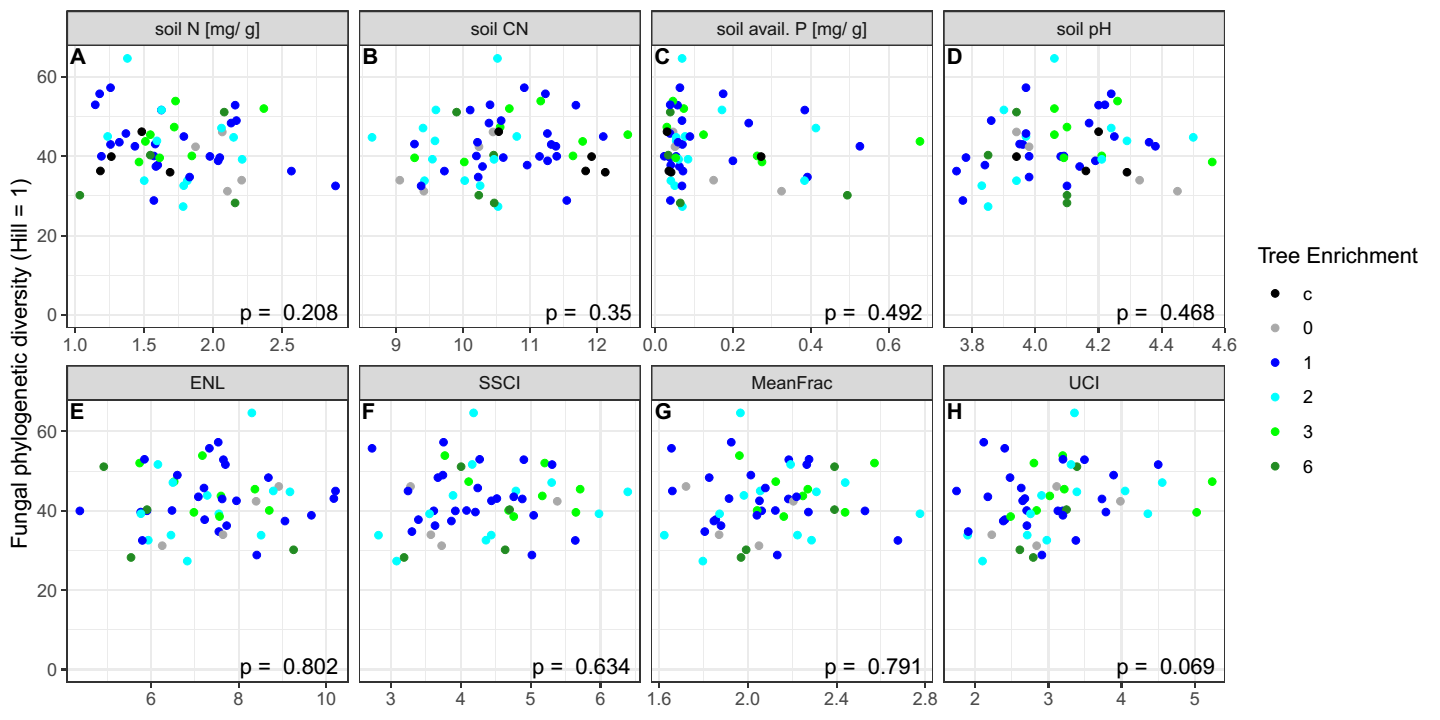


**Figure S4.** Relationship between fungal phylogenetic diversity (expressed as Hill number with dimension 1) and soil abiotic variables ((**A**): nitrogen, (**B**): carbon-to-nitrogen ratio, (**C**): available phosphorus, (**D**): pH), as well as different components of vegetation structural complexity ((**E**): effective number of layers (ENL), (**F**): stand structural complexity index (SSCI), (**G**): mean fractal dimension of polygons resulting from cross-sections in the point cloud (MeanFRAC), (**H**): understory complexity index (UCI)). N: nitrogen, C/N: carbon-to-nitrogen ratio, P: phosphorus. Linear models were fitted to the data No significant relationships (F test in ANOVA, p value < 0.05) were found. Colors denote tree richness; c: oil palm control plots with management as usual.


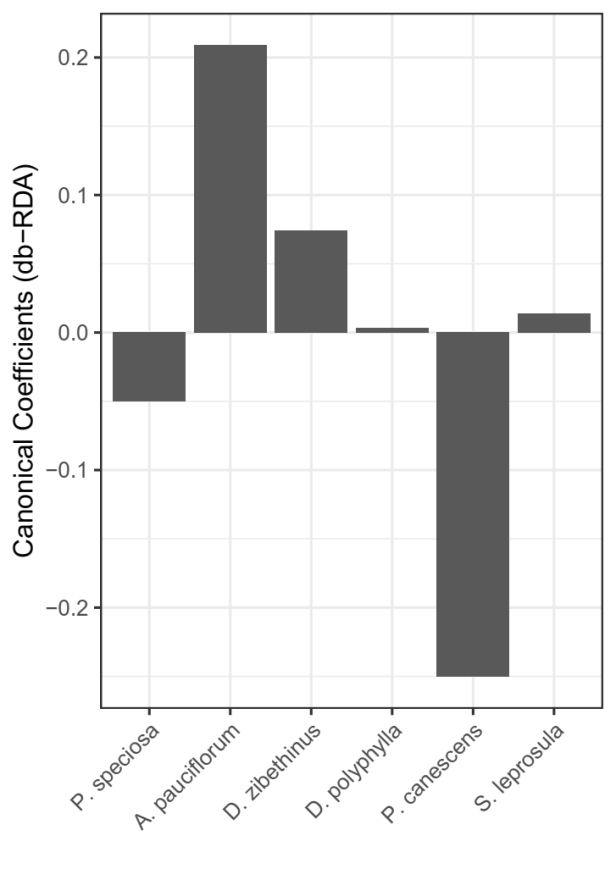


**Figure S5.** Canonical coefficients of distance-based redundancy analysis (db-RDA) associated with the effect of each planted tree species on the fungal community composition in the model for the random partitioning. A permutation test (999 permutations) was used to calculate statistic significances. No significant effects were found.

**Table 3.** Effect of soil variables and tree enrichment on fungal diversity in the oil palm plantation. Hill numbers were calculated with weight on OTU abundance equals zero (^0^D) and equals one (^1^D). Significant effects were tested using ANOVA. Outlier values of soil pH and nitrogen were replaced by their respective median values. Df : degree of freedom, Sq: square, CN: carbon to nitrogen ratio, p-value < 0.001 ‘***’, < 0.01 ‘**’, < 0.05 ‘*’. Non-linear tree richness is an approximation for interactions among tree species, independent of their identity.

|  | **Df** | **Sum Sq** | **Mean Sq** | **F-value** | **p-value** |
| --- | --- | --- | --- | --- | --- |
| *Taxonomic diversity (^0^D)* |  |  |  |  |  |
| Soil pH | 1 | 184,115 | 184,115 | 4.3511 | 0.04434* |
| Soil nitrogen [mg g^-1^] | 1 | 113,845 | 113,845 | 2.6904 | 0.10991 |
| Soil CN | 1 | 19,814 | 19,814 | 0.4683 | 0.4983 |
| Soil phosphorous [mg g^-1^] | 1 | 18,650 | 18,650 | 0.4407 | 0.51112 |
| Linear tree richness | 1 | 7,810 | 7,810 | 0.1846 | 0.6701 |
| Tree species identity | 5 | 425,863 | 85,173 | 2.0128 | 0.10094 |
| Non-linear tree richness | 3 | 124,029 | 41,343 | 0.977 | 0.41464 |
| Plot size | 3 | 49,061 | 16,354 | 0.3865 | 0.76341 |
| Residuals | 35 | 1,481,005 | 42,314 |  |  |
| *Taxonomic diversity (^1^D)* |  |  |  |  |  |
| Soil pH | 1 | 538 | 538.5 | 0.1825 | 0.6719 |
| Soil nitrogen [mg g^-1^] | 1 | 558 | 557.8 | 0.189 | 0.6664 |
| Soil CN | 1 | 1,671 | 1,670.7 | 0.5661 | 0.4568 |
| Soil phosphorous [mg g^-1^] | 1 | 4,995 | 4,994.5 | 1.6925 | 0.2018 |
| Linear tree richness | 1 | 3,355 | 3,355.1 | 1.1369 | 0.2936 |
| Tree species identity | 5 | 1,566 | 313.1 | 0.1061 | 0.9902 |
| Non-linear tree richness | 3 | 1,898 | 632.8 | 0.2144 | 0.8857 |
| Plot size | 3 | 6,241 | 2,080.5 | 0.705 | 0.5555 |
| Residuals | 35 | 103,285 | 2,951 |  |  |
| *Phylogenetic diversity (^0^D)* |  |  |  |  |  |
| Soil pH | 1 | 37,578 | 37,578 | 4.4764 | 0.04155* |
| Soil nitrogen [mg g^-1^] | 1 | 1,673 | 1,673 | 0.1993 | 0.65803 |
| Soil CN | 1 | 174 | 174 | 0.0207 | 0.88641 |
| Soil phosphorous [mg g^-1^] | 1 | 1,593 | 1,593 | 0.1898 | 0.66578 |
| Linear tree richness | 1 | 2,100 | 2,100 | 0.2502 | 0.62007 |
| Tree species identity | 5 | 76,999 | 15,400 | 1.8345 | 0.13154 |
| Non-linear tree richness | 3 | 27,708 | 9,236 | 1.1002 | 0.36204 |
| Plot size | 3 | 4,457 | 1,486 | 0.177 | 0.91129 |
| Residuals | 35 | 293,814 | 8,395 |  |  |
| *Phylogenetic diversity (^1^D)* |  |  |  |  |  |
| Soil pH | 1 | 44.31 | 44.313 | 0.5986 | 0.4443 |
| Soil nitrogen [mg g^-1^] | 1 | 134.84 | 134.835 | 1.8213 | 0.1858 |
| Soil CN | 1 | 91.72 | 91.719 | 1.2389 | 0.2733 |
| Soil phosphorous [mg g^-1^] | 1 | 125.83 | 125.829 | 1.6996 | 0.2008 |
| Linear tree richness | 1 | 11.87 | 11.874 | 0.1604 | 0.6912 |
| Tree species identity | 5 | 99.42 | 19.883 | 0.2686 | 0.9272 |
| Non-linear tree richness | 3 | 102.56 | 34.187 | 0.4618 | 0.7108 |
| Plot size | 3 | 101.58 | 33.86 | 0.4574 | 0.7138 |
| Residuals | 35 | 2,591.15 | 74.033 |  |  |
